# Long-range downstream enhancers are essential for Pax6 expression

**DOI:** 10.1016/j.ydbio.2006.08.060

**Published:** 2006-11-15

**Authors:** Dirk A. Kleinjan, Anne Seawright, Sebastien Mella, Catherine B. Carr, David A. Tyas, T. Ian Simpson, John O. Mason, David J. Price, Veronica van Heyningen

**Affiliations:** a MRC Human Genetics Unit, Western General Hospital, Edinburgh EH4 2XU, UK; b Genes and Development Group, Centres for Integrative Physiology and Neuroscience Research, University of Edinburgh, Hugh Robson Building, George Square, Edinburgh EH8 9XD, UK

**Keywords:** Pax6 gene regulation, Long-range enhancer, Transgenic mouse, Yeast artificial chromosome, Green fluorescent protein, Eye development, Paired-less isoform, Microphthalamia

## Abstract

Pax6 is a developmental control gene with an essential role in development of the eye, brain and pancreas. Pax6, as many other developmental regulators, depends on a substantial number of cis-regulatory elements in addition to its promoters for correct spatiotemporal and quantitative expression. Here we report on our analysis of a set of mice transgenic for a modified yeast artificial chromosome carrying the human PAX6 locus. In this 420 kb YAC a tauGFP-IRES-Neomycin reporter cassette has been inserted into the PAX6 translational start site in exon 4. The YAC has been further engineered to insert LoxP sites flanking a 35 kb long, distant downstream regulatory region (DRR) containing previously described DNaseI hypersensitive sites, to allow direct comparison between the presence or absence of this region in the same genomic context. Five independent transgenic lines were obtained that vary in the extent of downstream PAX6 locus that has integrated. Analysis of transgenic embryos carrying full-length and truncated versions of the YAC indicates the location and putative function of several novel tissue-specific enhancers. Absence of these distal regulatory elements abolishes expression in specific tissues despite the presence of more proximal enhancers with overlapping specificity, strongly suggesting interaction between these control elements. Using plasmid-based reporter transgenic analysis we provide detailed characterization of one of these enhancers in isolation. Furthermore, we show that overexpression of a short PAX6 isoform derived from an internal promoter in a multicopy YAC transgenic line results in a microphthalmia phenotype. Finally, direct comparison of a single-copy line with the floxed DRR before and after Cre-mediated deletion demonstrates unequivocally the essential role of these long-range control elements for PAX6 expression.

## Introduction

Gene regulation at the level of transcription requires the presence of a promoter, sometimes in conjunction with additional cis-regulatory elements such as enhancers and repressors. Developmental control genes form a particular class of genes with respect to their transcriptional regulation, as they often display highly complex spatiotemporal as well as quantitative expression patterns. Their expression needs to be induced or switched off at the right time and place in the embryo. Errors in the site, timing or levels of expression often lead to developmental malformations. To achieve such tightly controlled expression profiles many developmental control genes require the coordinated actions of a large number of cis-regulatory elements. These control elements can be spaced over large distances both upstream and downstream of the gene, and can even be located beyond or within introns of adjacent genes. Thus developmental regulators often reside in complex regulatory genomic landscapes (Kleinjan and van Heyningen, 2005). To achieve a better understanding of the genetic networks that govern the development and maintenance of our organ systems it is necessary to elucidate the molecular mechanisms controlling their transcription. As the cis-regulatory elements of developmental control genes form the hard-wiring of such networks in the genome (Davidson, 2001), their identification and characterization are an essential first step in this process. Pax6 is such a developmental regulator with many different functions during the development and maintenance of the eye, pancreas and central nervous system (Simpson and Price, 2002; Ashery-Padan and Gruss, 2001; Callaerts et al., 1997). A full characterization of the cis-regulatory elements required for Pax6 transcription in different tissues will not only be essential for studying the molecular mechanisms controlling its expression, but also greatly enhance the identification of the trans-acting factors binding to these elements, leading ultimately to a better definition of the regulatory networks that specify eye, brain and pancreas development.

Pax6 is a transcription factor with two DNA-binding domains. The paired domain located at the N-terminus is separated by a glycine-rich linker sequence from the second DNA-binding domain, the homeobox, followed by a proline–serine–threonine-rich transactivation domain at the C-terminus. It is highly conserved in very diverse range of species. In mammals, it is expressed in a complex spatiotemporal pattern during development of the retina, lens and cornea, in regions of the forebrain, hindbrain, cerebellum and spinal cord, the olfactory system and in pancreatic islet cells (Walther and Gruss, 1991; Stoykova and Gruss, 1994; Grindley et al., 1997; Quinn et al., 1996; St. Onge et al., 1997; Warren and Price, 1997; Dohrmann et al., 2000).

The importance not only of the correct spatiotemporal pattern, but also of the right level of expression is shown by the fact that haploinsufficiency for *Pax6* function (*Pax6*^*+/−*^) in the mouse results in the *Smalleye* (*Sey*) phenotype. Homozygotes (*Pax6*^*−/−*^) die perinatally with no eyes, no nasal structures and multiple severe brain abnormalities (Hogan et al., 1986; Hill et al., 1991; Stoykova et al., 1996; Caric et al., 1997; Warren and Price, 1997; St Onge et al., 1997; Mastick et al., 1997; Warren et al., 1999; Engelkamp et al., 1999; Pratt et al., 2000; Estivill-Torrus et al., 2001; Haubst et al., 2004). In humans, *PAX6* haploinsufficiency is the cause of the congenital eye malformation aniridia (Ton et al., 1991; Jordan et al., 1992; Glaser et al., 1992), and has more recently also been shown to cause brain defects (Sisodiya et al., 2001). Conversely, it was shown that overexpression of Pax6 in mice also leads to severe eye abnormalities (Schedl et al., 1996). In *Drosophila*, misexpression of the eyeless gene in imaginal disc primordia can lead to the formation of ectopic eyes (Halder et al., 1995). In the brain a *Pax6* gradient across the developing neocortex of mice is thought to be important for correct specification of its major areas (Bishop et al., 2000; Muzio et al., 2002). These findings imply that *Pax6* expression requires tight regulation and that different levels need to be maintained in different regions of the embryo.

Transcriptional control of Pax6 expression has been the subject of a number of studies (Plaza et al., 1995a,b; Williams et al., 1998; Kammandel et al., 1999; Xu et al., 1999; Kleinjan et al., 2001, 2004; Griffin et al., 2002; Kim and Lauderdale, 2006), resulting in the identification of several cis-regulatory elements through reporter assays in transgenic mice. While some of the cis-elements are found upstream and within introns of the gene, the presence of more, distant control elements downstream of the gene was brought to light through the analysis of human aniridia patients (Fantes et al., 1995a,b; Kleinjan et al., 2001). Whereas aniridia is generally caused by mutations or deletions that inactivate the protein product, a subset of aniridia patients was shown to have two intact copies of the PAX6 transcription unit. Instead some of these patients were found to carry chromosomal rearrangements with breakpoints in the 11p13 region downstream of PAX6 (Fantes et al., 1995b; Lauderdale et al., 2000; Crolla and van Heyningen, 2002), suggesting that the PAX6 promoters were separated from essential regulatory elements in the rearranged chromosomes. This view was reinforced by the analysis of human/mouse somatic cell hybrids with normal and rearranged patient chromosomes showing that expression from the rearranged chromosome was abolished (Lauderdale et al., 2000). The most distant patient breakpoint, SIMO, was located at 124 kb downstream from the PAX6 polyadenylation site. Analysis of the region beyond this breakpoint using YAC transgenic mice, DNaseI hypersensitivity mapping and reporter transgenic assays revealed the presence of several putative cis-regulatory elements, including ones driving expression in lens and retina (Kleinjan et al., 2001). These elements reside within introns of the adjacent, ubiquitously expressed ELP4 gene, but are nevertheless thought to be PAX6-specific long-range control elements (Kleinjan et al., 2002). Collectively these elements were termed the downstream regulatory region (DRR). Since then the availability of genomic sequence from a diverse range of species, enabling interspecies sequence comparisons, has demonstrated the presence of an unusually large number of evolutionarily conserved regions (ECRs) around the PAX6 gene. As ECRs are generally reliable indicators of the presence of cis-regulatory sequences, the presence of many more, as yet uncharacterized transcriptional control elements can be inferred.

One notable observation from the transgenic reporter studies described thus far is that several cis-elements drive expression with overlapping tissue specificity, suggesting that they may act via cooperative interactions in the endogenous locus. Such cooperative interactions are not taken into account in studies with plasmid-based reporter transgenics, which are also frequently hampered by position effects due to site of integration of the reporter cassette.

To study the long-range control of PAX6 expression, in particular the role of the DRR, we have generated transgenic reporter mice carrying one of two newly modified yeast artificial chromosomes (YAC) that report on the level of gene activation by production of GFP. Analysis of the pattern of GFP expression in a full-length single-copy line allowed us to demonstrate functional conservation between the human and mouse regulatory potential encoded in the full-length locus (Tyas et al., 2006). Here we describe the detailed analysis of six transgenic lines that vary in the extent of downstream PAX6 sequence that has integrated. Analysis of full-length and truncated YAC transgenes reveals the location and putative function of several novel tissue-specific enhancers. It also demonstrates that the absence of specific distal enhancers prevents expression of the reporter despite the presence of more proximal enhancers capable of driving expression in the same tissue. Complementary plasmid-based reporter transgenic analysis was used in some instances for detailed characterization of novel putative enhancers revealed by the YAC experiment. Comparative analysis, before and after Cre-mediated deletion, between a single-copy line carrying the YAC in which the DRR was flanked with LoxP sites, unequivocally demonstrates the essential role of these long-range control elements for PAX6 expression in some but not all tissues.

## Materials and methods

### Generation of the YAC transgenic reporter mice

The construction of YAC Y1123 has been described (Tyas et al., 2006). In short, a tau-GFP reporter followed by an IRES-neomycin resistance cassette was inserted in frame into the translation start site in exon 4 of the *PAX6* gene in a yeast targeting vector. This construct was recombined into YAC Y593 (Schedl et al., 1996) using URA3 as selectable marker, and the wildtype exon4, plasmid backbone and URA3 gene subsequently removed by counter selection in 5′ FOA to generate Y1123. A first loxP site was inserted into this YAC by homologous recombination of a LoxP-URA3-LoxP cassette at a position 140 kb downstream from the P1 promoter. The URA3 gene was removed by transient expression of Cre enzyme under control of the yeast GAL10 promoter. Finally a second LoxP site as well as a FRT site were inserted at a position 175 kb downstream from the P1 promoter using a targeting construct containing URA3, which was subsequently removed by 5′FOA counterselection. The resulting YAC was named Y374.

DNA was prepared for microinjection as described (Tyas et al., 2006), and microinjected into pronuclei from oocytes of F1 mice from a (C57/Bl6 × CBA) cross. Injected oocytes were replaced in pseudopregnant CD1 fosters (Hogan et al., 1994). Transgenic animals were identified by earclip analysis. All subsequent crosses were onto a CD1 background. Autoregulation effects were assessed by crossing transgenic line RBZ-146 with CD1 Pax6^S*eyEd*^*/+* mice, and by further crossing of a RBZ-146 transgenic heterozygous Pax6^S*eyEd*^*/+* male with a Pax6^S*eyEd*^*/+* female.

YAC transgenic lines were identified by PCR on DNA isolated from earclips according to standard procedures. Primers used to assay for the presence of the human PAX6 locus in the transgenic lines are

Pr025: 5′-CAAACAGGTTTAAAGACATTG-3′
Pr026: 5′-ACAGAGGTGCTTGTACAGAGT-3′

Primers used to detect the presence of the YAC arms:

YR1: 5′-ATATAGGCGCCAGCAACCGCACCTGTGGCG-3′
YR2: 5′-GTAATCTTGAGATCGGGCGTTCGA-3′
YL1: 5′-CACCCGTTCTCGGAGCACTGTCCGACCGC-3′
YL2: 5′-CCTTAAACCAACTTGGCTACCGAGA-3′

Primers used to determine the extent of the human PAX6 locus integrated in the various transgenic lines:

Pr028: 5′-CACTGTGCTTTTAAAAAGATAC-3′
Pr057: 5′-CCCATGGGCTACCTGTGC-3′
Pr126: 5′-TTACAATCTCCAGAGGTGGCA-3′
Pr127: 5′-TGTACTTAAACAGTGCTGGCA-3′
Pr128: 5′-ATGCCTCCATATCAACCTAGA-3′
Pr260: 5′-ACAGGGACACAACACTTCGCA-3′
Pr150: 5′-CCTATTCTTAAAATGTTTCTCTGT-3′
Pr151: 5′-AAGATCAGGCAGTAGGTAGGA-3′
Pr154: 5′-ATTTTGGTTCACAACGCCTTGCCT-3′
Pr155: 5′-GGCTTTCTATTTCTGGCTCTGCTG-3′
Pr267: 5′-CTGCGACAAGTTGGATGTGGT-3′
Pr268: 5′-ACTGGTGTATCTCTGATGGCT-3′
Pr363: 5′-TCACATACTTCCCTTTGCAAACCT-3′
Pr374: 5′-AGGACATAGCAGAGCTTTTGTGGA-3′
Pr375: 5′-AGGTGTTTAACATCCATTGGGAGA-3′
Pr399: 5′-TCTAGCACTATGCATCTTTAGGT-3′
Pr400: 5′-TATGAGCACGGAGTCTAATTAGG-3′

**Fig. 1:**
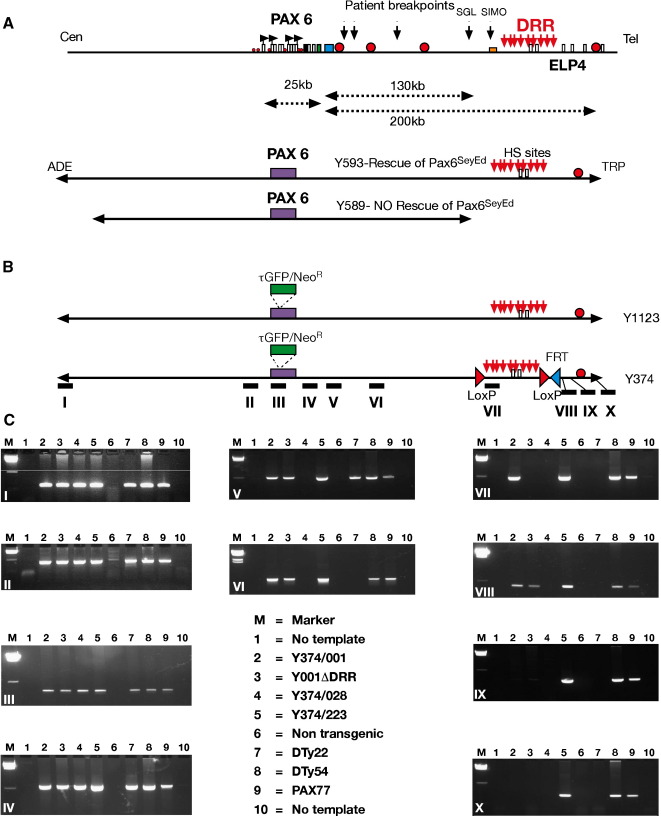
(A) Physical map of the human PAX6 locus. The relatively compact PAX6 transcription unit occupies a 25-kb fragment on human chromosome 11p13. Located downstream from PAX6 and transcribed in opposite direction is the ubiquitously expressed ELP4 gene. Blue rectangles indicate PAX6 exons, open rectangles ELP4 exons. Red circles indicate the positions of some significant enhancers. The positions of the breakpoints in some aniridia patients with a PAX6 position effect are indicated (Fantes et al., 1995a,b). Black horizontal arrows indicate known PAX6 promoters. Red vertical arrows show the position of a set of DNaseI hypersensitive sites (HS) that mark a region termed the PAX6 downstream regulatory region (DRR). The extent of the locus contained in the 420 kb YAC Y593 and the 310 kb Y589 is indicated below the physical map. Y593 extends 200 kb downstream from the final PAX6 exon and has been shown to rescue both heterozygous and homozygous *small eye* phenotypes (Schedl et al., 1996), while Y589 extending 125 kb downstream does not (Kleinjan et al., 2001). (B) Y593 has been modified by insertion of a tGFP-IRES-Neomycin-polyA-C2MAZ cassette (Tyas et al., 2006) in frame into the PAX6 ATG startcodon to generate YAC Y1123. Y1123 has been further modified to insert a LoxP site in a position just upstream from the SIMO patient breakpoint and a second LoxP site as well as an FRT site downstream from the furthest HS site, to generate a YAC, Y374, with a LoxP flanked region of 35 kb that includes the DRR. The positions of human-specific PCR primer sets used to characterize the extent of integration of the YACs in independent transgenic lines are indicated by roman numerals below Y374. (C) Gel photos of PCR fragments obtained with the primer sets indicated in panel B. All lines were positive for primer sets in the upstream and intronic region of the PAX6 locus, including additional upstream ones not shown here, but differed in the extent of downstream region that had integrated. M, marker; 1, no template; 2, line Y374/001; 3, line Y374/001ΔDRR; 4, line Y374/028; 5, line Y374/223; 6, non-transgenic mouse; 7, line DTy22; 8, line DTy54; 9, line PAX77 (Schedl et al., 1996).

They were used in the following combinations as shown in Fig. 1: Primer set I, YR1 and YR2; set II, Pr267 and Pr268; set III, Pr025 and Pr026; set IV, Pr 374 and Pr375; set V, Pr028 and Pr057; set VI, Pr399 and Pr400; set VII, Pr128 and Pr260; set VIII, Pr150 and Pr151; set IX, Pr154 and Pr155 and set X, YL1 and YL2.

For FISH analysis, spleens were dissected from transgenic mice, washed in PBS, punctured repeatedly with a sterile needle and splenocytes flushed out by forcing through 2 ml of RPMI. Lipopolysaccharide (LPS) was added and the culture incubated for 46 h. FISH was performed as described (Fantes et al., 1995a). Probes used were human cosmids cP60, located upstream from PAX6, cFAT5 (EMBL accession number Z95332) containing PAX6 and cosmids cC1170 (Z83306) and c11M20 (Z83308) located downstream from PAX6.

To study the expression patterns generated by the YAC transgenic mice embryos were dissected from pregnant females at E9.5, E10.5 and E17.5, taking the morning of the appearance of a vaginal plug as E0.5. Embryos were washed in PBS and examined and photographed immediately after dissection on a Leica MZ FLIII Microscope fitted with a Hamamatsu Orca-ER digital camera. For the E17.5 eye sections the eyes were removed from the embryo, washed in PBS and fixed overnight in 4% paraformaldehyde. Next morning the eyes were washed in PBS, and processed for sectioning on the cryostat. Sections were cut at 20 μ and counterstained with DAPI.

### Production and analysis of RB-Z reporter transgenic mice

The RB-Z reporter construct was made as follows: A 1.8-kb fragment was PCR amplified from cosmid c9A13 (EMBL accession number Z86001) using a high fidelity Pfu polymerase mix with PCR primers containing XhoI restriction sites. This fragment was subcloned into the SalI site of the p610+ reporter construct containing a Hsp68 minimal promoter-LacZ cassette as described for other PAX6 enhancers (Kleinjan et al., 2001). The microinjection fragment was isolated following digestion with NotI and Asp718I, and microinjected according to standard procedures. Transgenic mice and embryos were identified by PCR using LacZ-specific primers. Embryos were collected at the appropriate stages, washed in PBS and fixed for 1 h in a solution of 1% formaldehyde; 0.2% glutaraldehyde; 2 mM MgCl_2_; 5 mM EGTA and 0.02% NP-40 in PBS. After fixation the embryos were washed in PBS containing 0.02% NP-40, before being stained for several hours at 37°C in the dark in a solution containing 5 mM K_3_Fe(CN)_6_; 5 mM K_4_Fe(CN)_6_.3H_2_O; 2 mM MgCl_2_; 0.01% sodium deoxycholate; 0.02% NP-40 and 0.1% 5-bromo-4-chloro-3-indolyl-β-d-galactopyranoside (X-gal). Embryos were photographed on a Leica MZ FLIII Microscope fitted with a Hamamatsu Orca-ER digital camera with a CRI micro-color filter.

### RNA extraction and RT-PCR

RNA was isolated from dissected heads of E10.5 transgenic mouse embryos as well as non-transgenic littermates using TRI reagent (Sigma). The PAX6-expressing cell line CD5a was used as a control template for the human PAX6 locus. RT-PCR (33 cycles) was performed using AMV reverse transcriptase and random hexamers for first strand synthesis (Roche). Specific PCR primers used were:

Pr3F1675: 5′-GAGAGTGGACAGACATCCG-3′ (human exon 3 forward primer);
Pr αF: 5′-TCCTGGCGTCAATTTATCAGT-3′ (human exon α forward primer);
Pr050: 5′-TAGATCTATTTTGGCTGCTAGTC-3′ (reverse primer in exon 9);

The ubiquitously expressed ELP4/Elp4 gene (Kleinjan et al., 2002) was used as control for the RNA isolation and reverse transcriptase reaction. Primers used were:

Pr087: 5′-GCGACCGTCGGTGCGGAATGG-3′;
Pr088: 5′-AGAACATAGAGGAACTTGGTAAG-3′

## Results

### Generation of PAX6 reporter YACs and characterization oftransgenic lines

The two modified YACs used in this study are based on YAC Y593, which contains 420 kb of the human PAX6 coding sequence and flanking regions, extending beyond its putative regulatory elements. YAC593 has been shown to rescue the heterozygous eye phenotype and homozygous lethality of the mouse *smalleye* (*Pax6*sey/sey) phenotype, whereas a shorter YAC (Y589) containing 110 kb less flanking sequence did not (Fig. 1A) (Schedl et al., 1996; Kleinjan et al., 2001). From these results it was assumed that YAC593 contains all necessary cis-regulatory elements required for PAX6 expression. We have modified the YAC by introducing a tau-GFP and neomycin resistance cassette at the translational start site in exon 4, so that expression of the reporter cassette would be controlled by the PAX6 regulatory elements and would prevent the production of PAX6 protein from the YAC. This YAC was called Y1123 (Fig. 1B) (Tyas et al., 2006). The YAC has been further engineered to insert LoxP sites around the 35 kb long, distant downstream regulatory region (DRR) containing previously described DHS sites, to allow direct comparison between the presence or absence of this region in the same genomic location. Thus, a LoxP site was inserted at a position 145 kb downstream from the P1 promoter, about 3 kb upstream from the SIMO breakpoint. A second LoxP site, as well as an FRT site, was inserted 175 kb downstream from the P1 promoter, beyond the most distal DHS site mapped in Kleinjan et al., 2001. This YAC was called Y374 (Fig. 1B). Both YACs were used to generate transgenic mice. Five independent transgenic lines (two with Y1123: DTy22 and DTy54, and three with Y374: Y001, Y028 and Y223) were selected for further study.

**Fig. 2:**
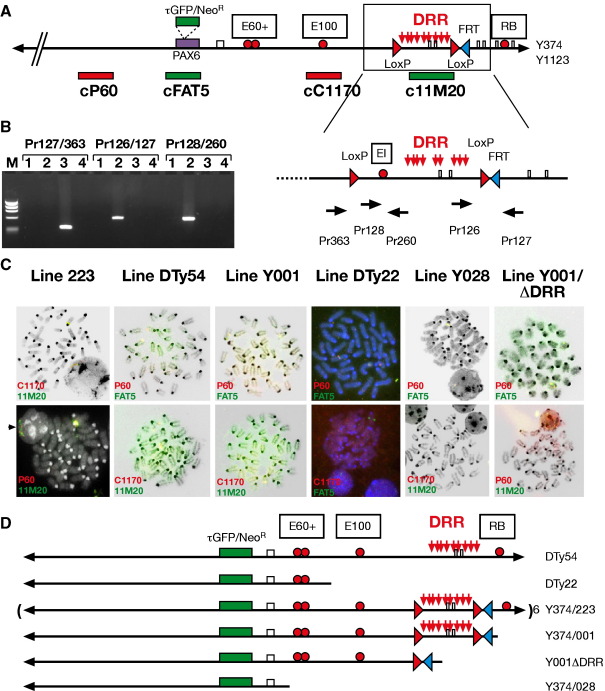
(A) Schematic map of YAC Y374 with the positions of the LoxP and FRT sites indicated. The PAX6 transcription unit is indicated by a purple box into which a tGFP-IRES-Neomycin cassette, indicated by a green box, was inserted. Red balls show the positions of some relevant enhancers for PAX6. Shown below the YAC are the positions of the cosmids used as probes in the FISH analysis of the transgenic lines. Cosmids P60 and C1170 were labelled with TexasRed and cosmids FAT5 and 11M20 with FITC. (B) Magnification of the DRR containing region of YAC Y374 flanked by LoxP sites. The positions of the PCR primers diagnostic for the Cre-mediated deletion are indicated. The gel photo shows the loss of the DRR region in the offspring of cross between a transgenic male of line Y001 and a germline Cre-expressing female. The newly established line was named Y001ΔDRR. After Cre-mediated deletion primers Pr363 and Pr127, which are 35 kb apart in parent line Y001, have come into close proximity and give a band of the expected size. PCR products spanning the distal LoxP/FRT site or recognizing a fragment within the DRR are no longer found in line Y001ΔDRR. M, marker; lane 1, no template; lane2, line Y001; lane3, line Y001ΔDRR; lane4, non-transgenic mouse. (C) FISH analysis on spleen cells from the individual transgenic lines. Cosmids used as probes were labeled in red (cP60 and cC1170) or green (cFAT5 and c11M20). All lines are positive for cP60, located upstream of PAX6, and for cFAT5, encompassing the PAX6 transcription unit. The variable extent of integration of the YAC is shown by the presence or absence of probes cC1170 and c11M20. Lines Y223, DTy54 and Y001 all contain both these probes, but DTy22 is negative for c11M20 and Y028 is negative for both c11M20 and c1170. Deletion of the DRR region from Y001 by Cre-mediated recombination has caused the deletion of the nearly full extent of cosmid c11M20 from the YAC transgene, and as a result Y001ΔDRR is negative for c11M20 signal. FISH signal from interphase nuclei of line Y223 shows it to carry 6 copies of the YAC arranged in a head to head, tail to tail order (arrowhead). The other lines are all single copy. (D) Summary of the YAC transgenic lines characterized in this study. Lines DTy54 and DTy22 are made with YAC Y1123. DTy54 is a single-copy full-length YAC insert, DTy22 is a single-copy line truncated around 100 kb downstream from the P1 promoter, just before a conserved sequence named E100. Y223, Y001 and Y028 are made by microinjection of YAC Y374, and Y001ΔDRR is derived from Y001 by Cre-mediated deletion of the DRR. Y223 carries 6 copies of the transgene including full-length ones. Y001 lacks between 10 and 20 kb from the distal downstream tip of the YAC including a highly conserved sequence element, RB. Y028 is severely truncated, terminating at 30 to 40 kb downstream from the P1 promoter, between ELP4 exon 10 and another highly conserved region termed E60+.

We first determined how much of the YAC had integrated in the transgenic lines, their copy number and whether the transgenes were intact or fragmented. PCR and Southern blotting with human-specific probes and primers was used to determine the extent of the YAC integrated in the mouse genome (Fig. 1C). Metaphase and interphase FISH on spleen cells from adult transgenic mice with cosmid probes derived from the human PAX6 locus was used to determine the copy number, whether the integrated YAC DNA was intact or fragmented across the genome, and to confirm the extent of YAC DNA present. The positions of the cosmids used as probes (cP60, cFAT5, cC1170 and c11M20) are indicated in Fig. 2A. The FISH results show that four lines, DTy22, DTy54, Y001 and Y028 are single copy. The fifth line, Y223, appears to have six copies of the YAC, arranged in a head to head and tail to tail sequence, based on analysis of several interphase nuclei (Fig. 2C). PCR analysis and FISH data with probes P60 and FAT5 indicate that all five lines contain the full extent of upstream sequence, including the left YAC arm. However, analysis of the region downstream from PAX6 indicates that only DTy54 and Y223 contain the full extent of the YAC, while DTy22, Y001 and Y028 are all truncated. The extent of DNA contained in these lines varies and is shown in Fig. 2D. Y001 lacks only a small region of between 10 and 20 kb from the very end of the YAC. DTy22 truncates between 50 and 75 kb downstream from the PAX6 P1 promoter, just before the E100 (previously called C1170/Box123) enhancer (Griffin et al., 2002). Y028 carries the shortest transgenic insertion, truncating between ELP4 exon 10 and a highly conserved sequence termed E60+ (Kleinjan et al., in preparation), thus containing the complete PAX6 coding region, but excluding nearly the entire downstream region.

### Analysis of GFP reporter expression in the YAC transgeniclines

**Fig. 3:**
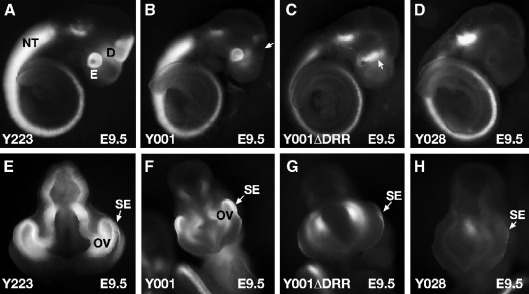
GFP expression in E9.5 embryos. (A) The GFP signal in Y223 reproduces the known Pax6 expression pattern with expression in the eye, telencephalon, diencephalon, rhombencephalon and spinal cord. (B) GFP expression is similar to panel (A) with exception of prosomere P2 of the diencephalon where expression is lacking in line Y001. (C) Deletion of the DRR results in loss of expression from prosomere P1 of the diencephalon as well as from the optic vesicle. Expression in prosomere P3 appears to be upregulated. (D) Truncated line Y028 shows expression in hindbrain and spinal cord, but completely lacks expression in forebrain and optic vesicle. (E) Frontal view of line Y223 shows expression in optic vesicle and surface ectoderm. (F) Line Y001 has expression in both optic vesicle and surface ectoderm, but (G) line Y001ΔDRR no longer expresses GFP in the optic vesicle while maintaining surface ectoderm expression. (H) A very weak surface ectoderm signal can be observed in line Y028. NT, neural tube; E, eye; D, diencephalon; OV, optic vesicle; SE, surface ectoderm.

**Fig. 4:**
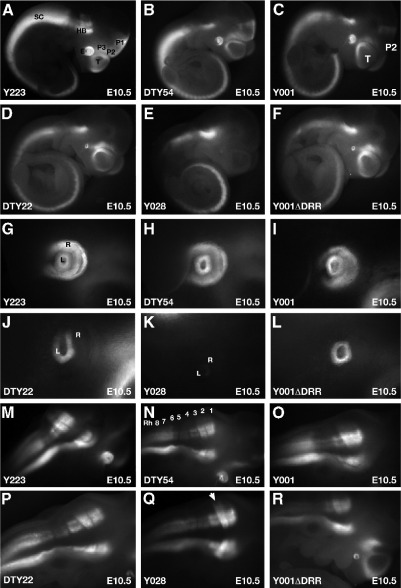
Expression in E10.5 embryos. (A, G, M) Line Y223 as well as (B, H, N) line DTy54 show a complete Pax6 expression pattern. (A, B) Expression is seen in the nervous system in telencephalon, diencephalon, rhombencephalon and spinal cord. (G, H) In the eye expression is found in lens and retina (M, N) in the hindbrain expression conforms to the known rhombencephalic pattern for Pax6. No expression is observed in Rh1 at this stage. Strong expression is found in three lateral columns in Rh2 and Rh3. Expression in Rh4-6 is weaker in two columns and lacking from the lateral most column. From Rh7 to caudal expression is strong and runs along the length of the spinal cord. (C, I, O) Expression in line Y001. (C) In the brain Y001 lacks expression in prosomere P2 of the diencephalon. (I) GFP signal is seen in both lens and retina. (O) Normal Pax6 expression in hindbrain. (D, J, P) In line DTy22 expression is absent in prosomeres P1 and P2 of the diencephalon. (J) No expression is found the retina, but (P) expression in hindbrain is normal. (E, K, Q) Line Y028 lacks expression in telencephalon and diencephalon. (K) No expression is seen in the eye apart from a very weak signal in the lens. (Q) Expression is found in spinal cord and rhombencephalon where the expression in Rh3 appears upregulated (F, L, R) Expression in line Y001ΔDRR needs to be compared to line Y001 (C, I, O). (F) GFP signal is seen in the brain in telencephalon and prosomere P3, but lacking in prosomeres P1 and P2. (L) Expression is present in the lens, but absent from the retina. (R) A normal expression pattern is found in hindbrain and spinal cord. SC, spinal cord; HB, hindbrain; E, eye; T, telencephalon; P1-3, prosomeres P1-3 of the diencephalon; L, lens; R, retina; Rh1-8, rhombomeres Rh1-8 of the hindbrain.

**Fig. 5:**
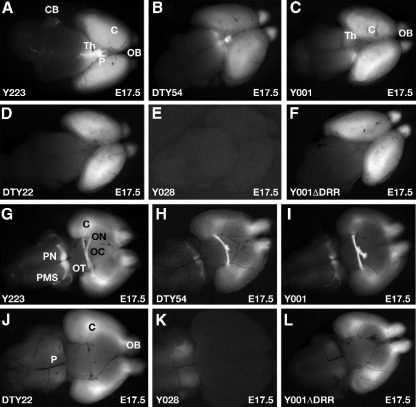
Expression patterns in E17.5 dissected brains, viewed from dorsal (A–F) and from ventral (G–L). (A) GFP signal in line Y223 is seen in the olfactory bulbs (OB), the cortical lobes (C), the pineal gland (P) and underlying thalamus (Th). (B) Expression in line DTy54 is identical to Y223. (C) In line Y001 expression is found in olfactory bulbs, cortex and thalamus, but absent from the pineal gland. (D) Line DTy22 has expression in cortex and olfactory bulbs, but no expression in thalamus and pineal gland. (E) The severely truncated line Y028 lacks all expression in the forebrain. (F) Deletion of the DRR region in Y001ΔDRR has resulted in loss of expression in thalamus and pineal gland, but expression in the olfactory bulbs and cortical lobes is maintained. (G) A ventral view of a Y223 brain shows a complex expression pattern with GFP seen in the olfactory bulbs and cortex, in the axons of the optic nerve (ON), through the optic chiasm (OC) and into optic tract (OT). Expression is also observed in the pontine region of the brain (P) with strong expression in the overlying pontine migratory stream (PMS) and the pontine nuclei (PN). (H, I) Expression in DTy54 and Y001 is as described for Y223. (J) In line DTy22 expression is seen in olfactory bulbs and cortex, but no longer observed in the optic nerve, chiasm and tract. Expression in the deeper regions of the pons (P) remains, but is no longer seen in the pontine migratory stream or in the pontine nuclei. (J) Line Y028 lacks all brain expression with exception of the pontine region where expression appears upregulated in comparison with the other transgenic lines. (L) In comparison with line Y001 line Y001ΔDRR has lost expression in optic nerve, chiasm and tract, as well as the pontine migratory stream and pontine nuclei. C, cortex; Th, thalamus; P, pineal gland; OB, olfactory bulbs; P, pons; PMS, pontine migratory stream; PN, pontine nuclei; ON, optic nerve; OC, optic chiasm; OT, optic tract.

We next examined the expression patterns of the GFP reporter in transgenic embryos collected at E9.5, E10.5, and E17.5. Expression of *τ*GFP in the full-length transgenic lines Y223 and DTy54 was identical to the known Pax6 expression pattern at all ages examined, with minor exceptions as described below. The expression patterns obtained from line Y223, carrying YAC374, and DTy54, carrying YAC1123, were identical at all stages, indicating that the insertion of the LoxP sites into YAC374 does not affect expression of the reporter cassette. The signal in Y223 embryos was generally stronger, in line with the higher copy number in this transgenic line. Thus, in both Y223 and DTy54 τGFP expression was observed in all tissues of the eye, including retina, lens and surface ectoderm, and in forebrain, hindbrain, spinal cord and olfactory system in a pattern mirroring endogenous Pax6 expression at E9.5, E10.5 and E17.5. Specifically, at E9.5 expression of the reporter was seen in telencephalon, prosomeres 1, 2 and 3 of the diencephalon, rhombomeres 2 to 8 of the hindbrain and caudally along the spinal cord. In the developing eye expression was observed in the optic vesicle and the overlying surface ectoderm (Fig. 3E). In E10.5 brains τGFP expression could be observed in telencephalon, diencephalon and rhombencephalon, as well as the spinal cord (Figs. 4A, B). In the eye expression was clearly visible in both lens and retina (Figs. 4G, H). The typical Pax6 expression pattern in the hindbrain was reproduced in the transgenic embryos, with expression absent in rhombomere 1 (Rh1) at this stage, strong expression in Rh2 and Rh3, weaker expression in Rh4-6, and stronger expression from Rh7 to caudal along the spinal cord (Figs. 4M, N). Later in development at E17.5 expression can be seen in the cerebral cortex, olfactory bulbs, ventral thalamus, pretectum, and the pineal gland (Figs. 5A, B), as well as in the pontine nuclei and pontine migratory streams and in the cerebellum (Figs. 5G, H). Expression in cerebellum was weak in comparison with the high endogenous expression level in this tissue (Engelkamp et al., 1999). Similarly, expression was detected in the olfactory epithelium (not shown), but at lower level than expected. The use of the τGFP reporter allowed cellular processes to be visualized. This effect is most clearly visible in the labeling of the axonal projections of retinal ganglion cells, which can be seen in the optic nerve and followed through the optic chiasm into the optic tract (Figs. 5G, H).

We proceeded to study the expression patterns generated in embryos carrying truncated versions of the reporter YACs. Line Y001 is a single-copy line lacking between 10 and 20 kb of the distal downstream tip of the full-length YAC sequence (Fig. 2D, see also Fig. 7). The pattern of GFP expression in this line showed a very similar expression pattern to lines Y223 and DTy54, with some notable differences. The first conspicuous difference from the full-length lines Y223 and DTy54 was the absence of expression in the P2 region of the diencephalon at E9.5 and E10.5 (Figs. 3B, F; 4C). Expression in the eye (Fig. 4I) and rhombencephalon (Fig. 4O) was normal. At E17.5 expression was similar to line Y223 and DTy54 (Figs. 5C, I) with exception of the notable absence of expression in the pineal gland (Fig. 5C).

Expression in line DTy22, truncated between 50 and 75 kb downstream from the PAX6 P1 promoter (see Figs. 2D and 7), was more restricted compared to line Y223. At E10.5 expression was similar to Y223 in telencephalon, rhombencephalon and neural tube (Figs. 4D, P), but different in the diencephalon with expression missing from both prosomeres P1 and P2, while expression in P3 appeared to be upregulated (Fig. 4D). In the eye expression was not observed in the retina, but clearly present in the lens (Fig. 4J). As in line Y001 expression was absent from the pineal gland at E17.5 (Fig. 5D). Expression in the thalamic region of the brain, still present in Y001, was not seen in line DTy22 (Fig. 5D). Furthermore, in the pontine region of the brain the distinct Pax6 expression pattern in the pontine nuclei and migratory streams is not observed, but signal is still seen in the underlying part of the pons (Fig. 5J).

Finally we examined GFP expression in line Y028. Y028 truncates between 30 and 42 kb from the P1 promoter, containing the full PAX6 coding region but lacking nearly the entire downstream region (Figs. 2D and 7). Expression in this line was severely restricted. At E9.5 expression was present in hindbrain and neural tube, but lacking entirely in the brain (Fig. 3D). No expression was seen in the eye with exception of a weak signal in the surface ectoderm (Fig. 3H). Similarly, at E10.5 expression could only be observed in the spinal cord and rhombencephalon (Fig. 4E). In the eye a very weak signal was seen in the lens, while expression in the retina was absent (Fig. 4K). Expression in rhombencephalon was normal, apart from an apparent upregulation of GFP signal in Rh3 (Fig. 4Q). The only expression seen in Y028 brains at E17.5 was in the interior part of the pons (Figs. 5E, K). All other, more rostral regions of the brain were negative for GFP. No signal was observed in the pontine nuclei or pontine migratory streams. However, expression of τGFP in the interior region of the pons appeared stronger in line Y028 relative to the expression level in line DTy22, suggesting a possible upregulation in this area in Y028.

As mentioned, expression in lines Y223 and DTy54 carrying the full-length YACs reproduced a nearly complete Pax6 expression pattern. However, some minor exceptions were noted. Firstly, the levels of expression in both olfactory epithelium (data not shown) and cerebellum at E17.5 were lower than expected. In particular the cerebellum expresses endogenous Pax6 at high levels in comparison with other tissues (Engelkamp et al., 1999). Although expression was clearly detectable in the multicopy line Y223, and at lower level also in lines DTy54 and Y001, this level of expression was very low in comparison with expression in other regions of the brain, e.g., the cortex or olfactory bulbs (Figs. 8A, B). This strongly suggests that additional cis-regulatory elements for expression in olfactory epithelium and the cerebellum exist that are located outside the region encompassed by the YAC. Alternatively tissue-specific post-transcriptional or post-translational differences between τGFP and Pax6 could exist. A second discrepancy between the τGFP pattern observed in our transgenic lines and the endogenous Pax6 expression pattern is that no expression could be detected in the pancreas of any of the lines at E11.5, E17.5 or P4. However, strong GFP expression was seen in the pancreas of older transgenic mice (> 3months) (not shown). This result was surprising as a recent report described the presence of pancreatic expression in transgenic mice carrying a Pax6 GFP reporter BAC containing a smaller portion of the Pax6 locus than our YAC transgenic lines (Kim and Lauderdale, 2006). Although unlikely, this could be due to a species difference as the BAC contains part of the murine Pax6 locus whereas our YACs contain the human PAX6 locus. However, the original PAX6-expressing YAC was able to rescue the Pax6 homozygous phenotype, suggesting that in the transgenic line made with this YAC expression in the pancreas was normal (Schedl et al., 1996; Heller et al., 2004). Thus far two enhancers have been described for Pax6 expression in the pancreas (Kammandel et al., 1999; Xu et al., 1999), located 4.2 kb and 2 kb upstream from the human P0 promoter respectively. We decided to check that the sequences of these enhancers were not mutated or deleted during the process of modifying the YACs. The pancreas enhancers were PCR amplified and sequenced with human-specific primers from genomic DNA of YAC lines Y223 and PAX77. Both sets of sequences were identical to each other and to the Genbank sequences for the regions (not shown).

### Analysis of RBZ reporter expression in transgenic lines

**Fig. 6:**
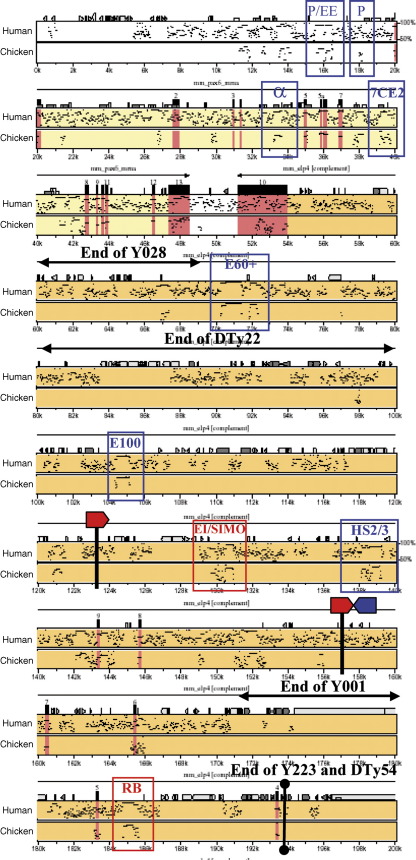
Annotated percentage identity plot (Schwartz et al., 2000) of the Pax6 locus extending from 20 kb upstream of the P0 promoter to the downstream end contained within the YACs. The murine sequence, being more compact than the human locus, was used as baseline. Pairwise comparisons were made with the human and chicken PAX6/Pax6 loci. The length of the Pax6 transcription unit is depicted in yellow and the Elp4 transcription unit in orange. Exons are shown in pink. The interval in which the truncated downstream end of the individual YAC transgenes lies is marked above the plot. The positions of the two LoxP sites inserted into YAC Y374 are indicated by vertical lines, with red and blue arrowheads marking LoxP and FRT sites respectively. Conserved enhancer sequences of relevance to this study are highlighted with blue boxes. P/EE, pancreas/ectodermal enhancer (Williams et al., 1998; Kammandel et al., 1999), P, pancreas enhancer (Xu et al., 1999), α, intron 4 retinal enhancer (Plaza et al., 1995a; Kammandel et al., 1999), 7CE2, diencephalon enhancer (Kleinjan et al., 2004); E60+, ultraconserved enhancer (Kleinjan et al., in preparation); E100, C1170box123 enhancer (Griffin et al., 2002), EI/SIMO, lens, diencephalon, hindbrain enhancer (Kleinjan et al., 2001), HS2/3, retinal enhancer (Kleinjan et al., 2001). Inspection of the PIP plot for the region between the 3′ ends of Y001 and Y223/DTy54 revealed the presence of one highly conserved fragment, which we have termed RB. The enhancer regions analyzed in Figs. 7 and 8, RB and EI, are marked with red boxes.

**Fig. 7:**
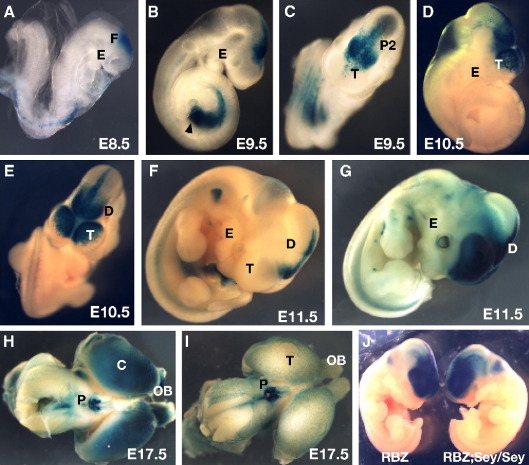
LacZ reporter expression in transgenic embryos for construct RB-Z. (A) In E8.5 embryos transgene expression is seen in the future forebrain region. (B, C) By E9.5 expression is seen in the diencephalic region of the brain, with strongest staining in the prosomere P2 region, extending in narrow line of expressing cells on either side of the midline through the P1 region and into the midbrain domain. In most lines some staining is also observed in the dorso-medial regions of the telencephalic bulges. The expression in the genital ridge is due to site of integration of the transgene. (D, E, F) At E10.5 and E11.5 expression remained strong in the P2 region of the diencephalon and increased in intensity in the telencephalic region in most (E, G), but not all (F), of the transgenic lines. (H, I, K) At E 17.5 the expression pattern driven by the RB element has become complex. All embryos show strong expression in the pineal gland. Most of the independent embryos also express in the cortical hemispheres, while in a smaller number of lines additional expression is seen in the olfactory bulbs. E, eye; F, forebrain; T, telencephalon; P2, prosomere P2 of the diencephalon; D, diencephalon; C, cortex; OB, olfactory bulbs; P, pineal gland.

As described line Y001 lacks between 10 and 20 kb of sequence from the very 3′ end of the insert compared to lines DTy54 and Y223 who both contain the full-length YAC. Y001 also lacks a subset of the expression pattern observed in lines DTy54 and Y223, notably expression is absent from the P2 region of the diencephalon at E9.5 and E10.5 and from the pineal gland at E17.5. To determine whether the region missing in line Y001 would contain tissue-specific enhancer(s) for prosomere P2 and pineal gland we examined a PIP plot of the region for conserved sequence elements (Fig. 6). One region of high conservation between the human, mouse and chicken sequences stood out in the 20 kb region at the distal end of the YAC insert. This element, which we have termed RB, has 94% conservation over 1100 bp between mouse and human, and is conserved to many species including chicken (87% over 835 bp), *Xenopus*, fugu and zebrafish. We have tested the enhancer capability of this element in transgenic mice using a minimal promoter-LacZ reporter cassette. Out of 20 independent transgenic offspring obtained at least 11 showed expression of the LacZ reporter gene. Six expressing lines were studied in detail at different embryonic stages. In E8.5 embryos transgene expression was seen along the midline in the forebrain region (Fig. 7A). By E9.0 expression was in the diencephalic region of the brain, becoming localized to the P2 region by E9.5 (Fig. 7B). A narrow line of expressing cells was also observed on either side of the midline in the P1 region spreading into the midbrain domain. Expression was also observed on either side in the dorso-medial regions of the telencephalic bulges (Fig. 7C). At E10.5 and E11.5 expression remained strong in the P2 region of the diencephalon and increased in intensity in the telencephalic region in most of the transgenic lines (Figs. 7D–G). At E 17.5-specific X-gal staining driven by the RB element can be observed in the pineal gland and the cortical hemispheres (Figs. 7H, I). In a number of independent lines additional expression is seen in the olfactory bulbs (Fig. 7H). Thus the lack of expression in prosomere P2 and pineal gland in YAC transgenic line Y001 can be attributed to the absence of cis-element RB (Table 1).

### The RB enhancer element is not Pax6-dependent

Previously we have described a cis-regulatory element in intron 7 of Pax6 capable of driving reporter gene expression in prosomeres P1 and P2 of the diencephalon. Expression driven by that element in the P2 region was shown to be under autoregulatory control, even though the endogenous gene remains transcribed in P2 in the absence of Pax6. This suggested the existence of an additional enhancer that would be active in the P2 region independent of Pax6 itself. Since the RB element drives expression in prosomere P2 we wanted to assess whether or not RB activity in P2 would be dependent on Pax6 functional activity. We therefore crossed the RB transgene onto the mouse *small eye* (Pax6^SeyEd^) background. A further cross with a *small eye* female resulted in a litter containing the RB-Z transgene on wildtype, heterozygous and homozygous Pax6^Sey^ backgrounds. Examination of E11.5 homozygous *small eye* mutant embryos carrying the transgene revealed the RB driven reporter expression pattern is identical to the RB pattern in wildtype embryos (Fig. 7J), indicating the RB element does not require functional Pax6 for its enhancer activity.

### CRE-mediated deletion of the DRR from YAC line Y001

A characteristic feature of transgenic experiments is the unpredictability of the transgene insertion site into the genome. It is well known that the local chromatin environment at the site of insertion can have a strong influence on the expression status of the transgene, thus obstructing a direct comparison between various independent transgenic lines inserted at random into the genome. Even though this is generally considered less problematic with large constructs such as YACs, we wanted to determine unambiguously the role of the downstream regulatory region (DRR) in regulating Pax6 expression. To enable a direct comparison between a DRR containing and a DRR lacking transgenic line we have flanked this region in one of our reporter YACs with LoxP sites (YAC374; see Fig. 1). Cre-mediated deletion of the DRR after establishment of a transgenic line would generate two transgenic lines that are genetically identical except for the presence or absence of the DRR. One of our transgenic lines, line Y001, proved most suitable for this experiment, and was crossed to a germ-line Cre-expressing line (line Cre745, containing a CAGGS-Cre construct in which Cre is under control of a chicken β-actin promoter (Araki et al., 1995)). The resulting transgenic line was called Y001ΔDRR, and tested genotypically to ensure that proper deletion of the region between LoxP sites had taken place. PCR analysis with primers on either side of both LoxP sites as well as with primers out with the deletion were used to show the removal of the 35 kb DRR region in line Y001ΔDRR (Fig. 2B). PCR with primer pair Pr126/127, located around the distal LoxP site gave the expected band in parent line Y001, but this band was no longer obtained in line Y001ΔDRR. PCR with Pr363, located just upstream of the proximal LoxP site, combined with Pr127, just beyond the distal LoxP, was negative in Y001 as these primers are 35 kb apart in this line, but positive in Y001ΔDRR as after the deletion these primers have been brought into close proximity (Fig. 2B). Finally, primer pair Pr128/Pr260, located within the DRR, produced a band from Y001 template, but not from Y001ΔDRR. The successful deletion of the DRR was further confirmed by FISH analysis on spleen cells derived from Y001 and Y001ΔDRR, using cosmid 11M20 as probe for the DRR region. The DRR constitutes nearly the full genomic insert of this cosmid and its deletion from line Y001ΔDRR results in an absence of FISH signal, whereas a clear signal is present in its parent line Y001 (Fig. 2C).

### Comparison of Y001 and Y001ΔDRR after deletion of the DRR

**Fig. 8:**
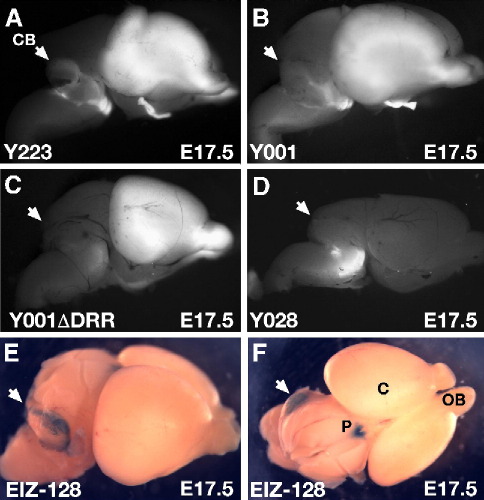
The DRR harbors an enhancer for expression in cerebellum. (A) Side view of an E17.5 brain of multicopy line Y223, showing expression in the cerebellum (white arrowheads). (B) In the single-copy line Y001 is weak but clearly present. (C) After deletion of the DRR in line Y001ΔDRR expression in cerebellum is no longer observed. (D) No cerebellar expression can be found in the truncated line Y028. (E) LacZ staining of a transgenic E17.5 brain for construct EIZ, containing two conserved elements located within the DRR, shows this enhancer is capable of driving expression in the cerebellum at this stage. (F) Additional staining is found in the pineal gland, identifying a second enhancer for pineal gland expression in the PAX6 locus. P, pineal gland; C, cortex; OB, olfactory bulbs.

Having confirmed the deletion of the DRR region we proceeded to examine the effect on expression of the reporter gene by comparison of Y001 and Y001ΔDRR embryos at E9.5, E10.5 and E17.5. At E9.5 and E10.5 line Y001 showed expression in the prosomere P1 region of the diencephalon, whereas expression was no longer seen at this site in Y001ΔDRR embryos (Figs. 3C and 4F). In the developing eye at E9.5 Y001 is expressed in the optic vesicle and surface ectoderm. While expression in surface ectoderm is maintained, expression in the optic vesicle is no longer observed after deletion of the DRR (Figs. 3C, G). Similarly at E10.5, when Y001 has strong expression in both lens and retina, Y001ΔDRR maintains lens expression but expression in the retina is completely lost (Fig. 4L). At E17.5 deletion of the DRR caused loss of expression in the thalamic region of the brain (compare Figs. 5C and F). In the eye, expression in the ganglion cell layer of the retina is lost, and as a result *τ*GFP is no longer seen in the optic nerve, chiasm and tract (Fig. 5L). In the pontine region of the brain the distinctive expression in the pontine migratory stream and pontine nuclei seen in Y001 is no longer present (compare Figs. 5I and L). Finally, the low level expression in the cerebellum of Y001 (Fig. 8B) is absent upon deletion of the DRR (Fig. 8C), suggesting a role for the DRR in driving cerebellar PAX6 expression.

**Fig. 9:**
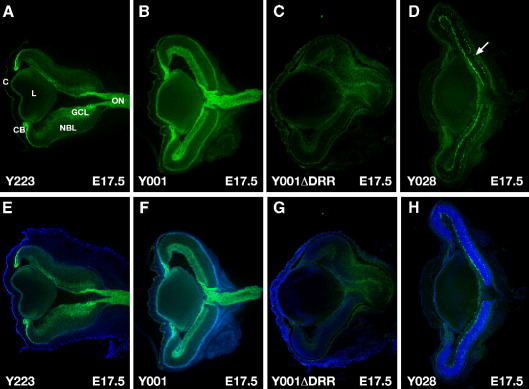
τGFP expression in E17.5 eyes on 20 μ cryosections. (A–D) *τ*GFP signal only (E–H) *τ*GFP plus DAPI. (A, E) Line Y223 shows expression in corneal epithelium, lens epithelium, iris, and ciliary body (CB). In the retina strong expression is seen in the ganglion cell layer (GCL) including the retinal ganglion (RGC) and amacrine cells, with weak expression in a subset of cells in the neuroblast layer (NBL). The presence of GFP in the axons of the RGCs gives rise to the strong staining in the optic nerve (ON). (B, E) Expression in line Y001 is the same as for line Y223, with GFP signal in corneal epithelium, lens epithelium, iris/ciliary body and the GCL and a subset of cells in the NBL of the neural retina. (C, G) Expression in line Y001ΔDRR is severely reduced in comparison with Y001, with GFP signal remaining in the corneal epithelium and very weakly in the GCL and NBL, but absent from lens, ciliary body and the RGCs. (D, H) Line Y028 shows similar expression as Y001ΔDRR, but relatively stronger expression in a cell layer within the GCL, most likely to be amacrine cells, and in a subset of differentiating neuroblasts in the NBL (white arrowhead). Note that panels C, D, G and H required 20 times longer exposure time than panels A, B, E, F. CB, ciliary body, C, corneal epithelium, L, lens, GCL, ganglion cell layer, NBL, neuroblast layer, ON, optic nerve.

We have also examined *τ*GFP expression in sections through E17.5 eyes. In the eyes from lines Y223 and Y001 the observed expression matches the known PAX6 expression pattern. *τ*GFP can be seen in the corneal epithelium, lens epithelium, iris and ciliary body. Expression is also seen in the neural retina, with strong expression in the ganglion cell layer (GCL), which at this stage contains retinal ganglion cells (RGCs) and amacrine cells, and much weaker staining in a subset of cells in the neuroblast layer (NBL) (Figs. 9A, B, E, F). The presence of *τ*GFP along the RGC axons generates a strong GFP signal in optic nerve. In both line Y001ΔDRR and line Y028 the *τ*GFP signal in the eye is severely reduced (Figs. 9C, D, G, H), as evidenced by a 20 times longer exposure time required for the photographs of the Y001ΔDRR and Y028 sections compared to those of Y223 and Y001 (Fig. 9). In line Y001ΔDRR GFP signal remains visible in the corneal epithelium and very weakly in some cells in the GCL and NBL, but has disappeared from the retinal ganglion cells, lens epithelium, iris and ciliary body (Figs. 9C, G). In the eyes of Y028 embryos expression mirrors that of line Y001ΔDRR, but surprisingly a relatively stronger *τ*GFP signal is seen in a layer of cells in the GCL and NBL, despite Y028 being a more severely truncated transgene (Figs. 9D, H). As no *τ*GFP is seen in the axons of the RGCs going into the optic nerve, the positive cells within the GCL are likely to be amacrine cells. GFP is also seen in a layer of differentiating neuroblasts within the NBL whose identity is under further investigation (Fig. 9D, white arrowhead).

In conclusion, removal of the downstream regulatory region from the YAC transgene as the only genetic difference between lines Y001 and Y001ΔDRR has resulted in multiple distinct differences in their expression patterns, demonstrating unequivocally the essential role of the DRR in driving expression in these regions.

### The EI enhancer region controls Pax6 expression in cerebellum

The observation that expression in the cerebellum was lost in line Y001ΔDRR led us to re-examine a transgenic line carrying the EI-Z construct. The EI-Z construct is a minimal promoter-LacZ reporter cassette under control of a 2.5-kb fragment encompassing the SIMO aniridia patient breakpoint region including two fragments of highly conserved sequence (Kleinjan et al., 2001). Reporter expression from this transgene has been described for early developmental stages (Kleinjan et al., 2001), but was not examined for later stages. At midgestation X-gal staining was seen in lens, diencephalon and in a specific pattern in the rhombencephalon, suggesting the possibility of expression in the cerebellum later in development. Indeed when expression was checked in E17.5 dissected brains staining was observed in the cerebellum (Fig. 8E). This suggests that the EI region, located 150 kb from the P1 promoter, controls at least in part expression of PAX6 in cerebellum. Reporter expression generated by the EI transgene was also found in the pineal gland (Fig. 8F), indicating that expression in that organ is under control of at least two enhancers, one located in the EI fragment and another in the RB fragment.

### Overexpression of a short PAX6 isoform causes microphthalmia

**Fig. 10:**
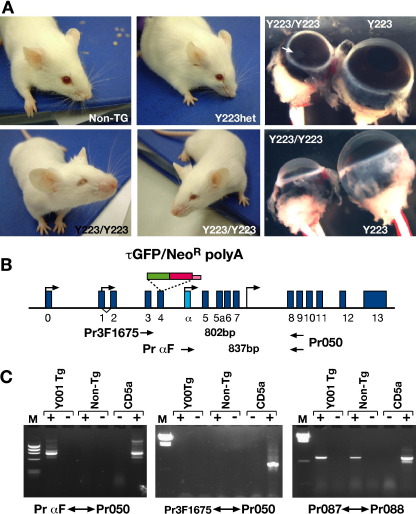
Overexpression of a short PAX6 isoform causes microphthalmia. (A) Mice homozygous for the 6-copy YAC transgenic line Y223 (thus carrying 12 copies of the transgene) have moderate to severe microphthalmia, sometimes in combination with cataracts. No obvious phenotype was seen in heterozygous or non-transgenic littermates. (B) Schematic map of the PAX6 transcription unit. PAX6 exons are shown as blue boxes. The tGFP-IRES-Neomycin-polyA reporter cassette was inserted into the canonical PAX6 translational start site in exon 4. Positions of known PAX6 promoters are indicated by arrows. Below the map the positions of PCR primers used in the RT-PCR reactions in panel C are indicated. Pr3F1675 is a human-specific primer in exon 3, primer aF is a human-specific primer in exon alpha, Pr050 is a reverse primer located in exon 9. (C) Gels showing the results of RT-PCR reactions with three primer sets on RNA made from the head region of an E10.5 Y001 transgenic embryo and a non-transgenic littermate. RNA made from the PAX6 positive human lens cell line CD5a is used as control. Lanes marked with + indicate where reverse transcriptase was added to the reaction, while − indicates where it was left out. Primer pair Pr087/Pr088, recognizing both human and mouse forms of the ubiquitously expressed ELP4/Elp4 cDNA are used as control for the RT reaction. No product is found with primers Pr3F1675 and Pr050 in line Y001 indicating transcription from promoters P0 and P1 is effectively stopped at the tGFP/Neomycin-polyA cassette. Primer pair PraF/Pr050 gave a band in Y001 cDNA demonstrating the presence of a shorter PAX6 transcript initiated from the Pα promoter. Both long (P0 or P1 derived) and short (Pα derived) isoforms are transcribed in the CD5a cell line.

The surprisingly low expression level in cerebellum, olfactory epithelium and the apparent absence of expression in the embryonic pancreas led us to attempt to further increase GFP signal strength in our strongest expressing line, the multicopy line Y223, by breeding this line to homozygosity. Unexpectedly this resulted in a strong eye phenotype in the homozygous offspring, whereas no obvious phenotype had been seen in the hemizygotes (Fig. 10A). The homozygous eye phenotype includes small eyes with abnormal iris, ectopic pigmentation, and retina, lens and ciliary body malformations and a high frequency of ectopic pupils (Fig. 10A). Closer inspection of hemizygous eyes revealed the occasional occurrence of ectopic pupils, but no reduction in eye size. As the τGFP-neomycin-polyA reporter cassette has been inserted into the PAX6 translational startcodon in exon 4 we had assumed to have knocked out PAX6 expression from the YAC transgene. However, in addition to the major PAX6 promoters P0 and P1 (Plaza et al., 1995a,b; Anderson et al., 2002), located upstream of exon 4, two less well-characterized promoters have been reported that are located downstream of the reporter insertion site in exon 4 (Kammandel et al., 1999; Kleinjan et al., 2001). Transcripts originating from those promoters would bypass the reporter cassette and give rise to a shorter PAX6 isoform lacking the paired domain (Fig. 10B). The existence of such an isoform has been observed in quail, as well as in a *C. elegans* Pax6 homologue (Carriere et al., 1993; Chisholm and Horvitz, 1995; Zhang and Emmons, 1995). At this time our observation was confirmed by a report describing a similar result in a multicopy BAC transgenic line where GFP was also inserted at the ATG in exon 4 (Kim and Lauderdale, 2006). In that study it was determined using a 5′-RACE method that during eye development an alternative Pax6 transcript originating from the alpha promoter in intron 4 is indeed made. No transcripts were found that initiate from the putative promoter in intron 7. We decided to determine whether this same transcript is also made in our reporter transgenic lines, using RT-PCR on RNA made from the heads of E10.5 transgenic embryos and non-transgenic littermates. As the YAC contains the human PAX6 locus, human-specific primers could be used to report only on reporter YAC derived transcripts. RT-PCR with primers 3F1675, specific for human exon 3, and reverse primer Pr050, located in exon 9, showed an absence of P0 or P1 derived transcripts in the E10.5 transgenic mouse embryo, while it is clearly present in the human control cell line CD5a (Fig. 10C) (Kleinjan et al., 2001). This demonstrates that the reporter cassette containing exon 4 does not get spliced out. RT-PCR between primer αF, specific for human exon α, and Pr050 detected the presence of a transcript of the expected size in the transgenic embryo, but not in a non-transgenic littermate, thus confirming the existence of Pα initiated transcripts in the heads of E10.5 embryos (Fig. 10C). Interestingly this transcript was also found in the CD5a control cell line. In combination with the report by Kim these results strongly suggest that the observed eye phenotype in the multicopy reporter transgenics is due to the overexpression of this shorter paired-less PAX6 isoform.

## Discussion

Developmental regulator genes occupy crucial positions in the genetic networks that control the development and maintenance of our organ systems, often fulfilling multiple functions at different places and timepoints in the embryo. This role requires very complex but strictly regulated control of gene expression. Thus many such genes reside in extended and intricate regulatory landscapes. Previous work on the regulation of PAX6 has shown that while it has a relatively compact transcription unit it requires a large intact genomic domain for proper expression and function (Kleinjan et al., 2001). Multispecies sequence comparisons of the Pax6 locus have revealed a large number of evolutionarily conserved regions (ECRs), not all of which have been characterized to date (see Fig. 7). Furthermore, the expression of reporter transgenics carrying small, isolated putative regulatory elements is subject to external effects due to site of integration as well as to the unnatural configuration of multicopy transgene arrays that is nearly always produced with small transgenes. Most importantly, such analyses neglect the potential interactions that occur between regulatory regions in the intact endogenous locus. To study the genomic requirements for correct Pax6 expression we have modified a yeast artificial chromosome (YAC) containing 420 kb of the human PAX6 locus by inserting a *τ*GFP-Neomycin reporter gene in frame into the main PAX6 translational start site. Previous analysis of transgenic mice made with the unmodified YAC had shown it to be capable of rescuing the phenotype of the Pax6 mutant *smalleye* (Pax6^*SeyEd*^), and it was therefore expected to carry most or all of the PAX6 regulatory domain (Schedl et al., 1996). Analysis of the *τ*GFP signal in two full-length transgenic lines indeed reproduced a complete Pax6 expression pattern, but with exception of expression in the developing pancreas. The absence of visible τGFP signal in the pancreas is puzzling since GFP was observed in transgenic mice carrying a Pax6 GFP reporter BAC containing a smaller portion of the Pax6 locus than our YAC transgenic lines (Kim and Lauderdale, 2006). While the BAC contains the murine locus whereas our YACs contain the human locus a species difference seems unlikely since the original PAX6-expressing YAC was able to rescue the Pax6 homozygous phenotype, suggesting that in the transgenic line made with this YAC expression in the pancreas was normal (Schedl et al., 1996; Heller et al., 2004). Thus far two enhancers have been described for Pax6 expression in the pancreas (Kammandel et al., 1999; Xu et al., 1999), located 4.2 kb and 2 kb upstream from the human P0 promoter respectively. If a mutation had been introduced in either of these during the process of modifying the YACs it could explain the discrepancy and furthermore potentially lead to identification of an essential binding site for Pax6 expression in the pancreas. However, sequencing of the enhancers in genomic DNA of modified and unmodified YAC transgenic lines Y223 and PAX77 respectively, revealed both sets of sequences to be identical to each other and to the Genbank sequences for the regions. One difference between the reporter used in the BAC mice and our YAC mice is our use of a tau-GFP fusion gene rather than GFP itself, leaving the possibility that the tau moiety causes instability of the reporter specifically in the pancreas. Further analysis will be required to resolve this issue.

Analysis of GFP expression in transgenic mice carrying full-length and truncated versions of the modified YAC has led to a number of conclusions. Firstly, comparison of the expression patterns generated in mice carrying complete and variously truncated versions of the YAC transgene has allowed the assignment of essential regulatory functions to specific intervals in the PAX6 downstream region. Using multispecies sequence conservation as a guide within these intervals we have subsequently homed in on putative cis-regulatory elements carrying out these functions by small element reporter transgenics. Using this approach we have identified a novel enhancer, which we have named RB, which drives expression in the P2 region of the diencephalon at midgestation and in the pineal gland at later stages. It also directs expression in the dorsal part of the telencephalon and in the olfactory bulbs in most, but not all independent transgenics. This enhancer is the most distal Pax6 enhancer identified to date located 210 kb downstream from the PAX6 P1 promoter.

The loss of expression in cerebellum upon deletion of the 35 kb DRR in line Y001ΔDRR revealed the putative presence of an enhancer for this tissue in the DRR region. We had previously made LacZ reporter transgenics with a fragment from within the DRR, construct EI-Z, and shown expression in a specific pattern in rhombencephalon at midgestation (Kleinjan et al., 2001), making one of the two highly conserved elements within the EI-Z construct a very good candidate to be a cerebellum-specific enhancer. Here we show that at E17.5 construct EI-Z does indeed drive expression in cerebellum. A third region harbouring elements of high sequence conservation is located in the region between the ends of the integrated transgene in lines DTy22 and Y028. This region contains an ultraconserved element (Bejerano et al., 2004) that we have termed element E60+. A detailed analysis of that element will be described elsewhere (Kleinjan et al., in preparation), but in summary it drives expression in telencephalon, the P3 region of the diencephalon (future ventral thalamus), the optic cup, the rostral migratory stream and olfactory bulbs, and the pontine migratory stream and nuclei, in accordance with the absence of expression in all these sites in Y028 embryos. Indeed, the very limited expression domain observed in transgenic line Y028 demonstrates the essential role of the whole downstream region for PAX6 expression.

A second conclusion that can be drawn from our study is that some enhancers appear to be absolutely essential for expression, while others may merely contribute to the overall level of expression in specific tissues. In some cases this could be due to those elements being the only enhancer for a particular expression site. For instance we show that the EI fragment carries an enhancer for expression in the cerebellum. Thus far it is the only known enhancer for PAX6 expression there; therefore it is unsurprising that its absence in line Y001ΔDRR (and DTy22 and Y028) results in absence of cerebellar expression in this line. However, the weak expression in cerebellum in the lines containing the enhancer strongly suggests the presence of another enhancer required for proper Pax6 expression in cerebellum, which must be located outside the genomic region contained in the YAC. Our experience gained through analysis of many cis-regulatory control elements situated around Pax6 has made clear that multiple enhancers exist for nearly every Pax6 expression site within the embryo. At present it is unclear how these regulatory regions cooperate to control Pax6 expression. To start to understand the molecular mechanisms involved will require the analysis of complete, full-length Pax6 loci with specifically directed mutations/deletions therein, either in the endogenous locus or in a YAC transgenic system as described here.

### The downstream regulatory region is essential for PAX6 expression

The use of CRE-mediated deletion of the Pax6 distal regulatory region (DRR) has allowed a direct comparison between a Pax6 locus with and without this region with the transgene at the same genomic integration site. A comparison of expression in the eye between lines Y001 and Y001ΔDRR highlights two opposing observations: Expression in the retina has been lost in Y001ΔDRR, while expression in the lens remains. A previous study has shown that the DRR contains a number of regulatory elements including a lens-specific element (the SIMO element, contained in the EI-Z construct) and a retina-specific element (located within a fragment containing HS2 and HS3 of the DRR) (Kleinjan et al., 2001). A number of other cis-regulatory elements are known to drive PAX6 expression in the developing lens including an element located upstream of the Pax6 P0 promoter, the ectodermal enhancer (EE) (Williams et al., 1998; Kammandel et al., 1999; Dimanlig et al., 2001). Thus it may be unsurprising that deletion of the SIMO element in the DRR has only limited effect on expression in the lens. It is difficult to estimate the decrease in the level of GFP expression in the lens, but the GFP signal in line Y001ΔDRR appears lower overall including in the lens. This result fits in with the analysis of mice with a targeted deletion of the ectodermal enhancer where Pax6 expression in the lens is decreased but not absent (Dimanlig et al., 2001). Similarly, multiple control elements are known to direct Pax6 expression in the retina, including the well-studied retinal enhancer in intron 4 of the gene (Plaza et al., 1995a; Kammandel et al., 1999). However, in contrast to the situation in the lens, deletion of the DRR, including the retina enhancer in the HS2/3 fragment, has completely abolished expression in the retina (see Figs. 4I, L). This demonstrates that the presence of the DRR is absolutely essential for Pax6 expression in the retina. Similarly, deletion of the DRR has abolished expression in iris and ciliary body in E17.5 eyes. The absolute requirement for the presence of the DRR (or at least some of the enhancers within it) can explain why the aniridia phenotype in ‘position effect’ patients is indistinguishable from aniridia in patients carrying coding region mutations in PAX6 (Crolla and van Heyningen, 2002; van Heyningen and Williamson, 2002).

Two more Pax6 expression sites, prosomeres P1 and P2 of the diencephalon show the same effect. The DRR contains an enhancer for expression in the prosomere P1 region of the diencephalon, located on the previously described EI-Z transgene (Kleinjan et al., 2001). Two further enhancers are known to drive expression in the same P1 region of the diencephalon, the CE2 element in intron 7 and the C1170Box123 element (renamed to E100 element) (Kleinjan et al., 2004; Griffin et al., 2002). Nonetheless, deletion of the DRR results in the total loss of expression in P1, demonstrating its essential role in Pax6 diencephalic expression. Similarly expression in P2 is directed through the intron 7 CE2 element and the RB enhancer, but absence of the latter results in loss of P2 expression. Unfortunately, the essential nature of the distal regulatory elements precludes any conclusions about the specific role of the more proximal elements. For instance, the E100 element (Griffin et al., 2002) lies within the interval between the ends of DTy22 and ΔDRR. Since no difference in GFP expression was observed between those two lines, the obvious, but incorrect conclusion would be that the interval between DTy22 and ΔDRR has no regulatory function. This highlights the value of studying individual cis-elements alongside larger transgenes.

Two scenarios that may account for the observations discussed above are: (1) there is a requirement for a direct interaction between multiple enhancers (and promoters) to set up a chromatin conformation that allows efficient Pax6 transcription to occur. In the absence of a crucial enhancer such conformation is not made leading to a failure to initiate transcription; (2) there is a requirement for the activity of the retina enhancer within the DRR before other retina enhancers can become active. In the absence of the ‘initiating’ activity subsequent enhancer function cannot take place. Further study, in particular the deletion of other retina enhancers in the context of the intact YAC or the endogenous locus, is required to distinguish between these mechanisms. An alternative experiment may be the deletion of the DRR using a Cre transgene under control of the intron 4 retina enhancer (Marquardt et al., 2001). If the DRR is required very early in development to activate the other retina enhancers, then its deletion by a ‘later enhancer’ driven Cre should give rise to continued expression of the GFP reporter in transgenic line Y001.

### Overexpression of a short PAX6 isoform causes microphthalmia

While breeding the multicopy line Y223 to homozygosity we noticed a distinct eye malformation, characterized by moderate to severe microphthalmia, in the homozygous offspring. This was surprising as we had inserted the reporter cassette with poly-adenylation sequence into the Pax6 translational start site in exon 4 of the gene, and included a C2MAZ sequence (Ashfield et al., 1994) to maximize the likelihood of transcriptional termination. A number of different Pax6 promoters have been identified, including the P0 and P1 promoters that generate transcripts encoding a full-length Pax6 protein containing a paired domain, homeodomain and PST-rich transactivation domain. Additional promoters have been identified in intron 4 (Pα) and intron 7 (P4) (Kammandel et al., 1999; Kleinjan et al., 2004), located beyond the reporter cassette insertion site. Transcripts initiated from these promoters would give rise to a shorter, paired-less isoform of Pax6. Such a protein has been described in quail (Carriere et al., 1993) and *C. elegans* (Chisholm and Horvitz, 1995; Zhang and Emmons, 1995). A similar observation of microphthalmia has been described recently in transgenics with multiple copies of a BAC that contains part of the murine Pax6 locus, and has a GFP reporter cassette inserted onto the ATG startcodon in exon 4 (Kim and Lauderdale, 2006). The authors show convincing evidence for the existence of Pα- but not P4-initiated transcripts in the eye and olfactory bulb. We have used RT-PCR helped by the fact that our YAC contains the human PAX6 locus, to confirm that a Pα derived transcript is made in our YAC transgenic mice, whereas no evidence was found to suggest that the reporter cassette containing exon 4 was spliced out in these animals. As the microphthalmia phenotype is observed in 3 independent multicopy lines (2 BAC and 1 YAC) it is unlikely to be caused by disruption of a gene at the transgene insertion site, but most likely to be the result of the overexpression of a paired-less PAX6/Pax6 isoform. Although initially no phenotype had been noticed in the 6-copy Y223 animals, we have subsequently observed a relatively high incidence of ectopic pupils in the hemizygous mice. In the case of the BAC transgenics an eye phenotype was observed with 16 or more copies, but not in animals with 8 or 10 copies, while in our case 6 copies gives a very mild phenotype and 12 copies a severe phenotype, possibly suggesting a stronger effect in the YAC. Interestingly the human lens derived cell line CD5a (Kleinjan et al., 2001) appears to express both P0/P1 initiated transcripts as well as the shorter Pα initiated transcript (Fig. 10) providing a useful source for further study into the role of this shorter PAX6 isoform.

### Gene control of complex developmental regulators

In conclusion, our study of full-length and truncated YAC transgenic mice has provided a wealth of information on the transcriptional regulation of PAX6. As shown here and in previous studies PAX6 has a large, extended control region containing many, complex cis-regulatory elements which interact in ways that are as yet poorly understood. Such complex transcriptional regulation involving large numbers of cis-elements is typical of many genes fulfilling complex roles as developmental regulators (Kleinjan and van Heyningen, 2005). The PAX6 gene is an excellent paradigm for this category of genes. The use of transgenic vectors with very large inserts (YACs or BACs) encompassing the (nearly) full gene locus is essential for the study of gene expression control for these developmental regulator genes. Combining these studies with small element transgenics provides a powerful approach to characterize the often highly complex interactions between multiple cis-elements required to drive the correct level of expression in a particular spatiotemporally defined pattern. Our study also highlights the need for a more complete view of the full regulatory code embedded in the gene locus, as multiple control elements may carry out overlapping and yet interdependent functions. Functional inactivation, through mutation, deletion or relocation, of any of these cis-elements can be a distinct cause of genetic disease.

## Figures and Tables

**Table 1:** Summary of reporter gene expression in full-length and truncated YAC transgenic lines

Line	E10.5								E17.5								
	SC	HB	T	P1	P2	P3	Ret	Lens	OB	C	Cb	ON	Pons	PMS	PN	Th	P
Y223	+	+	+	+	+	+	+	+	+	+	+	+	+	+	+	+	+
DTY54	+	+	+	+	+	+	+	+	+	+	+	+	+	+	+	+	+
Y001	+	+	+	+	−	+	+	+	+	+	+	+	+	+	+	+	−
Y001ΔDRR	+	+	+	−	−	+	−	+	+	+	−	−	+	−	−	−	−
DTY22	+	+	+	−	−	+	−	+	+	+	−	−	+	−	−	−	−
Y028	+	+	−	−	−	−	−	+/−	−	−	−	−	+	−	−	−	−

Abbreviations: SC, spinal cord; HB, hindbrain; T, telencephalon; P1, prosomere P1 of the diencephalon; P2, prosomere P2; P3, prosomere P3; Ret, retina; OB, olfactory bulb; C, Cortex, Cb, cerebellum; ON, optic nerve; PMS, pontine migratory stream; PN, pontine nuclei; Th, Thalamus; P, pineal gland.
